# Does Consumers’ Cultural Background Affect How They Perceive and Engage in Food Sustainability? A Cross-Cultural Study

**DOI:** 10.3390/foods13020311

**Published:** 2024-01-18

**Authors:** Julieth Lizcano-Prada, Marcela Maestre-Matos, Francisco J. Mesias, Olda Lami, Handan Giray, Celile Özçiçek Dölekoğlu, Abdou Gafarou Abdoulaye Bamoi, Federico Martínez-Carrasco

**Affiliations:** 1 Facultad de Ciencias Económicas y Empresariales, Universidad del Magdalena, Santa Marta 470004, Colombia; jlizcano@unimagdalena.edu.co (J.L.-P.); lmaestre@unimagdalena.edu.co (M.M.-M.); 2 Escuela de Ingenierías Agrarias, Universidad de Extremadura, 06007 Badajoz, Spain; fjmesias@unex.es; 3 Instituto Universitario de Investigación en Recursos Agrarios, Universidad de Extremadura, 06007 Badajoz, Spain; 4 Department of Agricultural Economics, Faculty of Agriculture, Eskisehir Osmangazi University (ESOGU), 26160 Odunpazarı, Türkiye; fatmahandan.giray@ogu.edu.tr; 5 Department of Marketing, Faculty of Business Administration, Adana Alparslan Türkeş Science and Technology University, 01250 Adana, Türkiye; cdolekoglu@gmail.com; 6 Department of Agricultural Economics, Institute of Natural and Applied Sciences, Akdeniz University, 07058 Antalya, Türkiye; aburohim0000@gmail.com; 7 Department of Applied Economics, Facultad de Economía y Empresa, Universidad de Murcia, 30100 Murcia, Spain; femartin@um.es

**Keywords:** food, consumer, cross-cultural, Spain, Türkiye, Colombia, sustainability

## Abstract

Motivated by the evolving global food landscape and its detrimental impacts on society, the environment, and health, this research aims to understand consumer perceptions, preferences and involvement regarding sustainable food products and consumption practices. To this aim, three countries were chosen for their distinct economic, cultural, and demographic differences (Spain, Türkiye, and Colombia), enabling an exploration of how these factors influence sustainability perceptions. The results show high levels of awareness, knowledge, consumption, and willingness to switch to more sustainable habits, although differences between countries were also found (price sensitivity in Spain and demand for information regarding sustainable food in Colombia). In addition, a group of consumers has been identified that is influenced by health, shows positive behaviours and perceptions towards sustainable food, and is not price sensitive. The study is significant, as it addresses the information gap between consumers, producers, and policymakers regarding sustainable food awareness. It seeks to provide insights into cultural influences on sustainability perceptions and aims to assist in developing educational programs and policies to promote sustainable consumption.

## 1. Introduction

Food production and consumption practices are continuously changing as a result of technological, economic and social developments. These include improvements in agricultural productivity, the increasing number of families with both partners working, and the demand for more convenient food. These changes have increased availability and access to food but have also had detrimental effects on society, the environment, and, in general, health [1]. Furthermore, some global events, particularly overlapped crises, have made these changes even more pronounced and challenging.

The world population reached over 8 billion as of 15 November 2022 according to the World Population Prospects Report [2], while the undernourished population increased from 8% to 9.8% between 2019 and 2021 [3]. Global population growth and, thus, the increasing demand for food have given rise to an agricultural system that relies primarily on intensive production with chemical inputs across vast areas of land, with food being later transported over great distances before consumption. This paradigm has produced both environmental damages—such as deforestation, soil depletion, loss of biodiversity, and pollution of rivers and groundwater [4]—and increasing greenhouse gas emissions, impacting climate change.

In addition, the rise in chronic non-communicable diseases [5,6] is also a result of the growing production and consumption of convenient and frequently ultra-processed food products [7] with high levels of sugar, salt, and fat content. This is why limiting the consumption of food products that are high in saturated fats, trans fats, salt, and sweets is included among the nutritional requirements of health organisations [7]. In this context, the increasing awareness of citizens regarding the preservation of the Earth, its ecosystems and biodiversity has given rise to the concept of sustainability, understood as “meeting society’s current needs without compromising the ability of future generations to meet theirs, ensuring a balance between economic growth, environmental stewardship and social well-being” [8].

As the current global food system cannot offset the negative impacts caused by these challenges, the international community has put more efforts into seeking sustainable food systems and the alternative food supply chains within them [9]. Such sustainable food systems are defined by FAO [4], as “those that deliver food security and nutrition for all in such a way that the economic, social and environmental bases to generate food security and nutrition for future generations are not compromised”.

Additionally, a worldwide commitment has emerged to develop more sustainable communities. This is represented, for example, by the United Nations Sustainable Development Goals (SDG) [10]. The importance of the food systems in this political initiative can be seen from the various SDGs which are related to food production and consumption and which reflect their importance for sustainability, such as SDG 2 “ending hunger, achieving food security and improving nutrition and promoting sustainable agriculture”; SDG 12 “to enhance sustainability by guaranteeing responsible consumption and appropriate production models”; and SDG 15 “to protect, restore and promote the sustainable use of terrestrial ecosystems, sustainably manage forests, contrast desertification and stop and reverse land degradation and halt biodiversity loss”. Despite the purposes stated in SDG 2 (ending hunger by 2030), and as stated before, the prevalence of undernourishment has started to increase again as of 2018 after remaining relatively unchanged since 2015 [3].

In response to these growing problems, the European Union (EU) has also taken strategic actions to stop or reduce the negative impact of human activity on the environment in the long-term through the European Green Deal (EGD), an integral part of the strategy developed by the EU to implement the UN Agenda for Sustainable Development. The EGD is expected to improve the well-being and health of citizens and future generations by providing various benefits, such as promoting healthy and affordable food through the Farm to Fork (F2F) strategy, sustainable farming and encouraging sustainable food consumption [11]. Within this context, it can be assumed that sustainability is conceived as a framework that must involve all stakeholders who can influence the system, from primary producers—including input suppliers—to final consumers and including waste management. In particular, one of the most important consensuses that have been achieved in recent years is the understanding that consumer choices, behaviours, lifestyles and consumption decisions play key roles in achieving sustainable development [12,13].

Various studies have analysed the behaviour of food consumers and their relationship with sustainability, although they have mainly focused on specific aspects or dimensions of sustainability [14,15,16,17]. However, there are fewer studies on sustainability as a global attribute and even more so if one takes into account the lack of a definition of this concept and the differences in perceptions of it amongst different geographical areas. Indeed, food choices, food purchases and food consumption can be strongly influenced by extrinsic factors (culture, religion, etc.) and intrinsic factors (income, development level of the country, etc.) [18,19].

Within this framework, food producers are increasingly interested in understanding the influence that sustainability as a concept has on consumers. Moreover, not only do they want to know about the level of awareness of individuals, but also the way in which this awareness is reflected in their purchasing and consumption behaviour, in what could be called their commitment to sustainability. It should be borne in mind that the concept of commitment in consumer behaviour can be defined as the preference towards a relation with a certain product/brand and company and the resistance to change [20].

Therefore, a survey was designed and conducted with food consumers in order to analyse their level of commitment to sustainability, as well as their perceptions of and preferences for more sustainable food products, production systems and consumption practices. Moreover, in order to reflect the intercultural differences that may affect these processes, this study has been carried out in three countries with different economic and cultural contexts. Hence, the main goal of this study is to gather cross-cultural consumer perceptions of and commitment to sustainable food consumption.

In order to do this, three countries—Colombia, Spain, and Türkiye—were selected to carry out the research. They are all highly unlike one another in terms of geography, culture, language, economic status, population, and even consumer spending, which makes them all clearly meet the study’s objectives. For instance, in Colombia, proximity to biodiversity may foster environmental awareness; in Spain, quality of life may drive preferences for sustainable products; and in Türkiye, the combination of traditions may influence attitudes towards nature. Economic availability and spending patterns also affect how sustainable practices are adopted and valued in each country, from more practical and affordable approaches to preference for higher-end products. For example, Spain, as a more economically developed country, might have a higher percentage of consumers willing to consume sustainable food products or services due to increased income.

In fact, this study showed very interesting and unexpected results, such as the higher perceptions and habits regarding sustainable purchasing and consumption among less developed countries (Türkiye and Colombia), as well as the presence of a price sensitivity among Spaniards, the country with the highest GDP. The results of the current study could therefore be useful not only to see if different cultural backgrounds affect consumers’ perceptions of sustainability, but also to narrow the information gap amongst farmers, the food business, and consumers on important topics for the agri-food sector, such as their awareness of sustainable food or which consumer groups are more likely to choose these products. Additionally, this study may help public institutions develop programs for educating people on sustainable consumption and policies to support producers and consumers.

The remainder of this paper is organised as follows: Section 2 details the data collection procedure and the methodology that has been followed to carry out this research. Subsequently, in Section 3 the paper presents the main findings and discusses them based on previous studies on the topic. Finally, Section 4 outlines the main conclusions of the paper, also presenting futures lines of research and recommendations to stakeholders.

## 2. Materials and Methods

### 2.1. Data Collection

The data analysed in this paper were obtained from a survey of 1000 individuals, which was carried out in three countries, namely Spain (324), Colombia (335) and Türkiye (341).

The research was approved by the University of Extremadura Bioethics and Biosecurity Committee (registration No. 1762022). The data were collected by means of a Google Forms questionnaire (www.docs.google.com), which was randomly distributed in several cities in Colombia, Türkiye, and Spain between October 2020 and April 2021 via social media and email. All participants were aged 18 or above and agreed to participate in the study, being assured that their answers would be kept confidential and completely anonymous. Respondents did not receive any compensation for their participation in the study.

The questionnaire included an initial section consisting of closed-ended questions regarding their level of awareness, knowledge and willingness to change their purchasing habits and consumption of sustainable food, for example, “Are you aware that the production and distribution of the food you consume has an environmental impact?”; “Do you know and have you ever consumed sustainably produced food products (SF)?”; “Do you think it is possible for you to change what and how you buy in order to make your habits more sustainable?”; and “Do you regularly consume sustainably produced food?”. These questions were followed by an open-ended question regarding the reasons behind why they do or do not purchase or consume sustainable food.

Given that it was assumed that some participants might be unfamiliar with the concept of sustainability, the following definition was previously presented: “Sustainability refers to meeting society’s current needs without compromising the ability of future generations to meet theirs, ensuring a balance between economic growth, environmental stewardship, and social well-being. There are several related concepts, such as environmental sustainability (which emphasizes the preservation of biodiversity without having to give up economic and social progress), economic sustainability (which seeks the profitability of activities in a sustainable manner) and social sustainability (which seeks population cohesion and stability)”.

Subsequently, self-assessment questions were asked regarding their level of agreement (Likert scale from 1 to 5) for a series of statements about sustainability in food consumption, taking into account their impacts at different stages (production, distribution, consumption and waste generation, etc.). This allowed us to quantify their importance for different segments of the population with increasingly sustainable consumption habits, and who would therefore form part of new consumer categories with a higher level of willingness to buy more sustainable products.

Finally, participants were asked questions regarding lifestyle and sociodemographic aspects. A pilot questionnaire was sent to 10 consumers (not included in the final sample) in order to ascertain the validity and clarity of the questions included in the study. Table 1 shows the sociodemographic characteristics of the sample for each of the countries.

### 2.2. Data Analysis and Segmentation

#### 2.2.1. Analysis of Open-Ended Questions

The open-ended questions of the questionnaire regarding the reasons behind their decision to consume or not sustainable food required a textual analysis of the answers for their categorisation and identification of dimensions. All valid words mentioned by participants were considered for data analysis. Firstly, the raw data were translated into English, as it was the common language of the research group. Then, a back-translation process [21] was applied for the words that were difficult to translate. This procedure was used to provide homogeneity in the coding process, i.e., in order to ensure that the same criteria were used to code data elicited in the three countries. The frequency of mention of each word was calculated for each country [22,23]. The phrases and words mentioned by participants were coded using the triangulation method [24]. First, a search for recurrent terms within each question was performed, and the terms with similar meanings were classified into categories, with the results presented in this paper being obtained by a consensus amongst the researchers to balance out the subjective influences of individuals [25,26]. The categories were merged into different dimensions using the same procedure. Considering the exploratory nature of the study, 5% was selected as a cut-off point to avoid missing a large amount of information [26,27]; therefore, the categories and dimensions mentioned by at least 5% of the consumers were considered for further analysis. Also taking into account the exploratory nature of the study, the frequency of mention was calculated regardless of whether the words were provided by the same participant or by different participants [26,28].

#### 2.2.2. Cluster Analysis

Cluster analysis was used in this document in order to allow a more in-depth study, identifying homogeneous subgroups of consumers that could reveal different perceptions towards sustainability. The inputs used were the various perceptions and levels of commitment towards sustainability.

Calculations were made using the cluster module of the IBM SPSS 21 statistical package, using a two-step procedure. Thus, and although a hierarchical cluster is frequently used in qualitative research [29,30], it was decided that the use of a combination of hierarchical and non-hierarchical (k-means) clustering was more convenient, as various authors recommend this mixed approach which allows the advantages of one method to compensate for the weaknesses of the other [31,32].

Firstly, a hierarchical clustering using Ward’s method was conducted using the abovementioned input variables. The final number of clusters was decided based on the agglomeration coefficient provided by SPSS [32], with two solutions with 3 and 4 clusters being obtained. Subsequently, K-means cluster analyses were carried out using the cluster centroids from the hierarchical analysis as the initial cluster seeds for the non-hierarchical procedure. Finally, the criteria used to decide on the final solution were based—as recommended by Hair et al. [32]—on the size of the clusters obtained, the significant differences between the clusters across the clustering variables, and the external validation through the interpretation of the clusters. Taking all these into account, a three-segment solution was finally selected. A variance analysis showed that all the segments differed significantly (*p* < 0.001) from each other with respect to the variables included in the analysis, which confirmed the validity of the results.

## 3. Results

### 3.1. Level of Environmental Awareness, Knowledge and Consumption of Sustainable Food Products

Table 2 reflects consumers’ awareness of the environmental impact of food production and distribution; their knowledge of sustainable food products, and their willingness to switch to more sustainable purchasing habits.

From the analysis of Table 2, it can be observed that the majority of the participants in this study (96.7%) reveal a high level of awareness of the environmental impact resulting from food consumption and distribution, with minimal differences amongst the countries under analysis. However, the general levels of knowledge and consumption of SF are relatively low among all countries. These findings are in line with some studies, such as that of WWF and Sancho [33], but are in disagreement with other authors, such as Hartmann et al. and Quoquab and Sukari [34,35].

It is worth mentioning that Spain ranks first in terms of awareness, knowledge, consumption, and willingness to change what and how they buy in order to make their habits more sustainable. Interestingly, Turkey is the country that most believes that this change has a low level of environmental impact compared to other sectors. Nonetheless, only less than 5% of the participants refuse to change their purchasing habits. These results are also found by Martínez-Carrasco and Prado [36], revealing a trend of consumers shifting towards more sustainable food.

Studies such as that of Wang et al. [37] strongly suggest that developed-economy countries hold international leadership in sustainable consumption and production practices, in line with the outcomes of this study, which notably align with the economic statuses of the countries involved. Spain stands out with the most advanced economy, boasting a gross national income adjusted for purchasing power parity of $40,910 per capita per year [38], followed by Türkiye ($30,290) and, lastly, Colombia ($16,540).

Moreover, WWF and Sancho [33] suggest that apart from the economic situation in Colombia, other factors can strongly influence their diet, such as tradition and the indigenous culture.

After participants were asked whether they consumed sustainable food, they were requested to state the reasons behind their choices. Table 3 presents several determining factors that were highlighted. As for Spain, the main reasons that were refraining from consuming SF were the higher prices and lack of accessibility. As one of the Spanish participants stated:

*“I know perfectly well that choosing organic products helps improve our environment and local economy, that even the taste of food is much better and healthier, but unfortunately, our economy does not allow me to do so. With the current salaries, feeding a family of four with sustainable food becomes a luxury that is not within the reach of the vast majority of citizens.”*

Interestingly, this was not the case for Türkiye and Colombia, which, in comparison to Spain, are considered poorer countries according to their GDP. As a matter of fact, this positive attitude in developing countries towards willingness to pay more for a more sustainable product has been previously reported in other studies, such as that of Mostafa [39].

Notably, the lack of information is the main barrier among Colombia and Turkey, in line with conclusions from Quoquab and Sukari [35] indicating that developing countries are comparatively far behind developed ones regarding the adoption and practice of sustainable consumption because of their lack of knowledge, possibly as a result of increasing advertising and promotion of unsustainable and ultra-processed food products [40,41].

Lack of information has also proven to be a very important barrier in other studies, such as those of Eldesouky et al., Feucht and Zander, and Quoquab and Sukari [15,35,42]. Other relevant factors that were frequently mentioned by most consumers were the fact that they believed they could not have access to these types of food near their homes or that they were produced in very small quantities, this being an important feature also found by Zanoli and Naspetti [43] when studying organic food purchase motivators. Moreover, recent studies in Colombia explain that the main reasons why Colombian consumers do not consume sustainable food are price and limited access to this type of food [33,44].

What stands out is the significant percentage that mention price as the main barrier in Spain, the country with the highest GDP. However, the low relevance of this factor in the other countries may be due to their low knowledge of these type of products and, consequently, the lack of information on their prices.

On the other hand, the main reasons behind the consumption of sustainable food were coherent among all three countries, with the awareness of the participants being first. The main statements within this category were related to contributing to a more sustainable environment and helping the local economy and local producers. Additionally, respondents thought of these type of food products as healthier options with higher quality, due to the fact that they perceived SF principally as more natural, less processed, and therefore, safer and more nutritious. These factors have also been found by Zanoli and Naspetti [43] where consumers had a significant interest in “natural” products and healthy products.

### 3.2. Sustainable Behaviours in Food Consumption

Later, consumers were presented with various statements regarding their perceptions and habits regarding sustainable food purchasing in terms of its environmental, socioeconomic, and health impacts, as well as the importance given to price when making purchasing decisions. Consumers were requested to score each statement from one to five, with results being shown in Table 4.

The results from Table 4 reveal moderate to high scores for sustainable statements, and a moderate score for the importance given to price. It is noteworthy in this cross-cultural study that highly significant differences are found in all statements regarding perception and habits regarding sustainable food purchasing across countries. The results show that, generally, there is a high tendency toward sustainable habits and perceptions in all countries that participated in the study, with a slightly higher rating given to environmental sustainability self-assessments.

Interestingly, Türkiye is the country that generally gave the highest scores overall, followed by Colombia and Spain. Remarkably, Spaniards only stand out on the higher importance they give to the use of reusable bags. Meanwhile, Colombians score the highest on the positive impact on employment/wealth generated when purchasing local; the negative impact of meat consumption on sustainability; the consumption of unpackaged food; the belief that vegetarian diets are more sustainable; and affordable prices ensured by intensive food production. Finally, Turkish consumers gave more importance to most of the statements included in this study concerning both socioeconomic sustainability and environmental sustainability, while also giving the lowest importance to price.

Particularly, most of the Colombian consumers agree on the association between meat production and environmental pollution, a fact that coincides with studies such as those by Blanco-Murcia and Ramos-Mejía [45] and WWF and Sancho [33], where consumers closest to meat production areas were more sensitive to this fact and, consequently, tended to reduce their meat consumption to once a week on average.

A strong preference for sustainable diets and responsible consumption practices among Colombians was also found by Idárraga-Tunjo et al. [46]. Their consumption habits are defined as much by health or well-being as by family tradition, which means that the way they cook food is linked to a transfer of knowledge and that their eating habits are conditioned by the way they were educated [33]. This tradition is accompanied by the farmer heritage; it is part of the connection that people make with nature and food production. In that sense, most of the Colombian consumers state that it is very important to buy local and support local farmers [33].

Turkish consumers, on the other hand, granted higher scores to health and environmental statements, findings in line with other studies such as that of Çakmakçı [47].

### 3.3. Consumer Segmentation with Respect to Distinct Dimensions of Sustainable Habits and Perceptions

Table 5 presents the scores given by the three groups of consumers to the different statements about sustainable habits and perceptions. As can be seen, a first group of respondents (Cluster 1) was identified, with 34.5% of the population, which includes individuals who are not very concerned about sustainability in the purchasing and consumption of food and have a low perception of the negative impact that intensive production systems have on the environment. This is the group of citizens with the lowest scores in almost all statements. It is also worth mentioning that the environmental impact of modern production and the consideration of more natural food products as healthier had very low ratings, with a significant difference in comparison to Cluster 2 and Cluster 3. This cluster, therefore, has been named “Less-concerned consumers”.

The second group identified, with 30.0% of the sample, is the group with the highest ratings in social–economic dimensions and lower ratings in statements regarding the impact of meat consumption on the environment. It is worth mentioning that this cluster is the least concerned group when it comes to price, which is why they have been called “Price indifferent–health concerned consumers”.

Cluster 3, with 35.5% of the participants, presents a higher level of sensitivity towards the impact of their consumption and habits on the environment. The ratings given by these consumers to almost all these items were between four and five, which means a high level of agreement or commitment with the statements regarding their purchasing decision and consumption habits. However, in this Cluster 3, more relevance is given to the fact that the current production systems guarantee food at affordable prices. For all the above, this group has been called “Price sensitive–environmentally driven consumers”.

The second stage of the analysis was carried out in order to identify relevant sociodemographic data (Table 6) and the relationship between clusters and consumer awareness, knowledge, and willingness to consume sustainable food products (Table 7).

This analysis allowed us to identify a significant relationship between the different assessments made on sustainability in food consumption and the sociodemographic characteristics.

Thus, “Less-concerned consumers” are predominantly males, younger, and mostly Spanish participants. These patterns are found in other studies among the Spanish population, such as that of Gutiérrez-Villar et al. [48], where less sustainable dietary patterns were found among the youngest consumers.

“Price indifferent–health concerned consumers” have higher levels of socio-economic sustainability in consumption—mostly health statements—and are characterised by a majority of females, middle-aged and young-middle-aged people with university education and higher incomes (which can explain the price insensitivity of this group), and a majority of Turkish people. In another study, Özenoǧlu et al. [49] found that the healthy eating attitudes of Turkish people are at high and ideal levels. These healthy lifestyles and diets in Türkiye are positively correlated with sustainable food consumption according to previous studies, such as those of Gürler And Özkaya et al. [50,51]. Additionally, significant relationships between sociodemographic variables and sustainable behaviours were found in Türkiye [51] and in other worldwide studies, such as those of [52,53,54,55]. Moreover, a study on the effect of demographic variables on price sensitivity of customers found that age and gender can impact price sensitivity, with middle-aged and female consumers being less price sensitive [56].

On the other hand. “Price sensitive–environmentally driven consumers” are characterised by being mostly young women with the lowest income and mainly composed of Colombians. Research into Colombian consumer attitudes toward sustainable food indicates that economic factors such as product costs and price fluctuations in eco-friendly goods significantly impact their choices, leading them to favour purchasing locally sourced food products [57].

In Table 7, the results are in line with the findings showed in the previous table, with “Less-concerned consumers” being the group with the lowest level of knowledge and consumption of sustainable food products, also with the highest percentage of consumers that are unwilling to change their habits. On the other hand, “Price indifferent–health concerned consumer” had the highest levels of awareness, knowledge, consumption and willingness to change their consumption habits to even more sustainable ones.

## 4. Conclusions

This paper has attempted to provide cross-cultural insights into consumers’ involvement in food sustainability, their perceptions and behaviours. A number of findings from this study can be emphasised, namely, the unexpectedly high positive scores in terms of sustainable awareness, knowledge and consumption and, most importantly, the consumers’ high level of willingness to change habits towards more sustainable ones, despite their beliefs that this change might have only a low level of impact on overall sustainability.

This study has found significant differences amongst the analysed countries, with Türkiye being the country with the highest ratings in assessments related to sustainable purchasing and consumption habits and perceptions, followed by Colombia and, lastly, Spain, the country with the highest GDP. Another interesting finding was the `price insensitiveness in Turkey, considering that their GDP is much lower than that of Spain. Nevertheless, there is a strong need to raise awareness and increase accessibility to SF, especially in Colombia, which might explain why price was not ranked as the main factor. These results initially confirm the effect of culture, in its diverse facets, on the perception of sustainability, and allow us to assume that similar differences to those found here will appear in other countries around the world.

Furthermore, the consumer segmentation carried out allowed us to identify two segments of citizens who are more prone to sustainable consumption. One of the clusters, whose members are price indifferent and concerned about health, might be a very good target group, due to its high level of willingness to change towards more sustainable purchasing and consumption habits and their lack of price sensitivity.

The findings of this study may help narrow the gap between farmers, food businesses, and consumer in terms of sustainability and sustainable food consumption, as well as help public institutions develop programs for educating people on these types of food products.

Certainly, a larger and more representative sample in terms of sociodemographic characteristics would have been preferable for this study; however, this was difficult to achieve due to the allocated resources and the lack of willingness to respond to the survey. Therefore, more in-depth studies are recommended concerning the anomalous findings.

This study must be understood as an exploratory type of research, due both to the sampling method used and the characteristics of the sample; therefore, its conclusions may not be directly generalised. It is necessary to carry out further quantitative studies with wider and more representative samples in order to confirm and expand our findings. Nevertheless, the results obtained can be extrapolated to other countries with similar characteristics to the ones under analysis in this paper, with a view to develop future research and plan marketing actions.

## Figures and Tables

**Table 1:** Sociodemographic data for the sample (%).

		Spain	Colombia	Türkiye	Total
Sex ^n.s.^	Male	42.0	49.6	48.7	46.5
	Female	58.0	50.4	51.3	53.5
Age ***	18 to 34 years old	43.8	86.0	29.0	52.9
	35 to 50 years old	25.6	9.3	45.5	26.9
	>50 years old	30.6	4.8	25.5	20.2
Studies ***	Primary education	12.0	0.9	4.1	5.6
	High School/Vocational Training	27.8	75.8	52.5	52.3
	University Degree	60.2	23.3	43.4	42.1
Family income ***	Low	52.5	89.3	7.6	49.5
	Medium	27.5	9.3	35.2	24.0
	High	20.1	1.5	57.2	26.5
Family members ***	1–2	26.30	9.00	22.60	19.70
	3–4	73.80	91.00	64.30	75.60
	>4	0	0	13.20	4.70

Significance: *** *p* < 0.01, ^n.s.^—not significant.

**Table 2:** Level of awareness, knowledge and willingness to consume Sustainable Food (SF) %.

	Awareness ^a^ **		Knowledge ^b^ ***		Consumption of SF ^c^ ***		Willing to Change Purchasing Habits ^d^ ***		
	No	Yes	No	Yes	No	Yes	No	Yes^willing to change^	Yes^low impact^
Spain	2.8	97.2	36.7	63.3	50.9	49.1	4.3	66.7	29.0
Colombia	3.9	96.1	50.1	49.9	52.8	47.2	1.8	55.8	42.4
Türkiye	3.2	96.8	47.2	52.8	50.7	48.4	6.5	40.5	53.1
Total	3.3	96.7	44.8	55.2	51.4	48.5	4.2	54.1	41.7

^a^ Are you aware that the production and distribution of the food you consume has an environmental impact? ^b^ Do you know, and have you ever consumed sustainably produced food products (SF)? ^c^ Do you regularly consume sustainably produced food? ^d^ Do you think it is possible for you to change what and how you buy in order to make your habits more sustainable? Answers: No; Yes; Yes, but I consider that it has a low level of impact since there are other sectors that have a much higher environmental impact (industry, transportation, etc.) Significance: ** *p* < 0.05 *** *p* < 0.01.

**Table 3:** Determining factors on decisions regarding consumption of sustainable food %.

		Spain	Colombia	Türkiye
Reasons why they do not consume sustainable food products (%)	Price	51.56	9.66	14.29
	Lack of trust	4.69	1.38	4.76
	Lack of information	9.38	68.28	28.57
	Interest	4.69	6.21	9.52
	Not easily accessible	20.31	9.66	23.81
Reasons why they consume sustainable food products (%)	Health	25.71	21.90	30.50
	Quality	15.10	12.06	24.82
	Environmental and Social Awareness	48.57	44.76	31.91
	Taste	6.53	3.17	2.84

**Table 4:** Consumer self-assessment of perceptions and habits regarding sustainable purchasing and consumption.

		Spain	Colombia	Türkiye	Total
Socioeconomic sustainability	I try to buy local and national products for the employment/wealth generated ***	4.08	4.25	4.25	4.19
	Intensive food production assures affordable prices, which is my main concern ***	2.77	2.94	2.33	2.67
	I follow a balanced diet because I am concerned about the effect of food on my health ***	4.26	3.89	4.38	4.18
	I try to buy vegetables produced in a more natural, organic way… because I believe they are healthier ***	3.36	3.89	3.92	3.73
	Meat from extensive livestock farming can contribute to the development of rural areas and the preservation of the environment ***	3.90	3.76	4.09	3.92
Environmental sustainability	I try to consume unpackaged or bulk food products ***	4.01	4.13	3.67	3.93
	I try to buy local and national products because of the impact of transportation **	3.88	4.03	4.07	4.00
	I try to buy food produced in a traditional way and that contributes to the preservation of the environment ***	3.72	3.87	4.05	3.88
	I limit my food purchases to what I need, and reuse food products ***	4.50	4.30	4.58	4.46
	I actively recycle at home ***	4.15	3.92	4.17	4.08
	Meat consumption negatively impacts sustainability***	3.27	3.49	2.59	3.12
	Modern production has a mayor environmental impact ***	3.34	3.78	3.96	3.70
	Vegetarian diets are more environmentally sustainable than diets including animal origin food products ***	2.80	3.29	2.83	2.97
	I try to buy seasonal food ***	3.65	3.39	4.08	3.71
	I try to use reusable bags because of the negative impact of plastic bags ***	4.46	4.31	4.01	4.26

Significance: ** *p* < 0.05, *** *p* < 0.01.

**Table 5:** Segmentation by sustainable habits and perceptions.

		C1 (*n* = 345)	C2 (*n* = 300)	C3 (*n* = 355)	Total (*n* = 1000)
Socioeconomic sustainability	I try to buy local and national products for the employment/wealth generated **	3.53	4.58	4.51	4.19
	Intensive food production assures affordable prices, which is my main concern ***	2.85	1.87	3.18	2.67
	I follow a balanced diet because I am concerned about the effect of food on my health ***	3.74	4.53	4.30	4.18
	I try to buy vegetables produced in a more natural organic way…because I believe they are healthier ***	2.83	4.31	4.10	3.73
	Meat from extensive livestock farming can contribute to the development of rural areas and the preservation of the environment ***	3.59	4.28	3.93	3.92
Environmental sustainability	I try to consume unpackaged or bulk food products ***	3.45	3.97	4.38	3.93
	I try to buy local and national products because of the impact of transportation *	3.23	4.34	4.45	4.00
	I try to buy food produced in a traditional way and that contributes to the preservation of the environment ***	3.11	4.39	4.20	3.88
	I limit my food purchases to what I need and reuse food products ***	4.10	4.71	4.60	4.46
	I actively recycle at home **	3.63	4.30	4.32	4.08
	Meat consumption negatively impacts sustainability ***	2.76	2.26	4.19	3.12
	Modern production has a mayor environmental impact ***	2.78	4.22	4.14	3.70
	Vegetarian diets are more environmentally sustainable than diets including animal origin food products ***	2.75	2.09	3.94	2.97
	I try to buy seasonal food ***	2.97	4.05	4.14	3.71
	I try to use reusable bags because of the negative impact of plastic bags ***	3.88	4.45	4.46	4.26

Significance: * *p* < 0.1, ** *p* < 0.05, *** *p* < 0.01.

**Table 6:** Relationship between clusters and participants’ sociodemographic variables (%).

Second Stage of the Analysis		“Less-Concerned Consumers” (C1)	“Price Indifferent–Health Concerned Consumers” (C2)	“Price Sensitive–Environmentally Driven Consumers” (C3)	Total
Sex ***	Male	53.4	45.1	41.2	46.5
	Female	46.6	54.9	58.8	53.5
Age ***	<35 years old	58.3	43.3	55.8	52.9
	35 to 50 years old	21.4	37.0	23.7	26.9
	>50 years old	20.3	19.7	20.6	20.2
Studies **	Primary education	7.8	4.3	4.5	5.6
	High School/Vocational Training	52.8	45.0	58.0	52.3
	University Degree	39.4	50.7	37.5	42.1
Family income ***	Low	54.2	33.0	58.9	49.5
	Medium	23.2	25.0	23.9	24.0
	High	22.6	42.0	17.2	26.5
Family members ^n.s.^	1–2	17.1	20.1	21.9	19.7
	3–4	79.5	73.5	73.6	75.6
	>4	3.4	6.4	4.5	4.7
Country ***	Spain	41.4	24.7	34.0	32.4
	Colombia	35.5	18.5	46.0	33.5
	Türkiye	27.0	46.3	26.7	34.1

Significance: ** *p* < 0.05, *** *p* < 0.01, ^n.s.^—not significant.

**Table 7:** Relationship between clusters and consumer awareness, knowledge and willingness to consume SF.

	Awareness ^a n.s.^		Knowledge ^b^ ***		Consumption of SF ^c^ ***		Willing to Change Purchasing Habits ^d^ *			Purchaser ^e^ **		
	No	Yes	No	Yes	No	Yes	No	Yes^low Impact^	Yes^willing to change^	Never	Sometimes	Yes. Always
C1	3.5	96.5	52.4	47.6	63.4	36.6	5.3	43.0	51.8	5.5	52.4	42.1
C2	2.2	97.8	40.4	59.6	43.9	56.1	2.2	39.6	58.3	5.8	47.8	46.4
C3	4.2	95.8	43.4	57.0	47.1	52.9	3.9	43.1	53.1	2.9	32.8	64.3
Total	3.2	96.8	45.3	54.7	51.6	48.3	3.7	41.7	54.5	4.9	45.3	49.9

^a^ Are you aware that the production and distribution of the food you consume has an environmental impact? ^b^ Do you know And have you ever consumed sustainably produced food products (SF)? ^c^ Do you regularly consume sustainably produced food? ^d^ Do you think it is possible for you to change what and how you buy in order to make your habits more sustainable? Answers: No; Yes; Yes, but I believe that it has a low level of impact since there are other sectors that have a much higher environmental impact (industry, transportation, etc.). ^e^ Do you usually do the food purchasing? Significance: * *p* < 0.1, ** *p* < 0.05, *** *p* < 0.01, ^n.s.^—not significant.

## Data Availability

The data presented in this study are available on request from the corresponding author. The data are not publicly available due to privacy and ethical restrictions.
